# Composition of cutaneous bacterial microbiome in seborrheic dermatitis patients: A cross-sectional study

**DOI:** 10.1371/journal.pone.0251136

**Published:** 2021-05-24

**Authors:** Martijn G. H. Sanders, Tamar Nijsten, Joost Verlouw, Robert Kraaij, Luba M. Pardo

**Affiliations:** 1 Department of Dermatology, Erasmus Medical Centre, Rotterdam, The Netherlands; 2 Department of Internal Medicine, Erasmus Medical Centre, Rotterdam, The Netherlands; Skin Research Institute Singapore, SINGAPORE

## Abstract

**Background:**

Seborrheic dermatitis (SD) is a chronic inflammatory skin disease with a multifactorial aetiology. *Malassezia* yeasts have been associated with the disease but the role of bacterial composition in SD has not been thoroughly investigated.

**Objectives:**

To profile the bacterial microbiome of SD patients and compare this with the microbiome of individuals with no inflammatory skin disease (controls).

**Methods:**

This was a cross sectional study embedded in a population-based study. Skin swabs were taken from naso-labial fold from patients with seborrheic dermatitis (lesional skin: n = 22; non-lesional skin SD: n = 75) and controls (n = 465). Sample collection began in 2016 at the research facility and is still ongoing. Shannon and Chao1 α- diversity metrics were calculated per group. Associations between the microbiome composition of cases and controls was calculated using multivariate statistics (permANOVA) and univariate statistics.

**Results:**

We found an increased α-diversity between SD lesional cases versus controls (Shannon diversity: Kruskal-Wallis rank sum: Chi-squared: 19.06; global p-value = 7.7x10^-5^). Multivariate statistical analysis showed significant associations in microbiome composition when comparing lesional SD skin to controls (p-value = 0.03;R^2^ = 0.1%). Seven out of 13 amplicon sequence variants (ASVs) that were significantly different between controls and lesional cases were members of the genus *Staphylococcus*, most of which showed increased composition in lesional cases, and were closely related to *S*. *capitis S*. *caprae* and *S*. e*pidermidis*.

**Conclusion:**

Microbiome composition differs in patients with seborrheic dermatitis and individuals without diseases. Differences were mainly found in the genus *Staphylococcus*.

## Introduction

Seborrheic dermatitis (SD) is a common chronic relapsing inflammatory skin disease occurring mainly on the sebum-rich areas of the face, scalp and chest. The disease spectrum is heterogeneous ranging from a mild form of scaling confined to the scalp to severe erythema and scaling of the scalp, face and trunk. The aetiology of SD is complex. Host factors such as skin barrier dysfunction, genetic susceptibility and immune status may all influence disease risk [1–3]. Also, several life style and environmental factors increase risk for SD. In recent epidemiological studies this has been shown for male gender, white skin colour, winter climate, generalized dry skin and specific diets [4, 5].

Since the condition was first described in 1887 [6], it is often assumed that overgrowth of species of the genus *Malassezia* cause and maintain SD. The *Malassezia* yeast are more abundant on affected skin, and treatments targeting this yeast (e.g. ketoconazole) can effectively treat SD [7]. However, the genus *Malassezia* is also part of the healthy microbiome and similar species of *Malassezia* in SD patients and controls have been found. There might be a causal relationship between *Malassezia* and SD, but it is not the only factor [8]. A few more recent studies have shown that next to a higher abundances of *Malassezia* yeast, SD patient might also have bacterial dysbiosis on the scalp [9, 10]. One other study investigated the microbiome on the face of 24 patients with SD and found a higher density of *Acinetobacter*, *Staphylococcus* and *Streptococcus* in lesional skin when compared with non-lesion skin [11]. As this study did not include healthy controls and these bacteria are also part of the normal skin microbiome, it is not clear whether they are indeed associated with SD. Also, members of the *Staphylococcus* genus can act as pathogens and thus it would be of interest to investigate which specific species are relevant in SD. In addition, as for any observational studies, initial findings need to be replicated in an independent cohort.

As stated above, most studies have focused on the role of *Malassezia* or were based on small series of cases with no controls, which are necessary to control for ‘normal’ variation in the microbiome. Here we profiled the bacterial microbiome of the nasolabial fold of participants with SD in a middle aged and elderly population based study in the Netherlands (Rotterdam Study) and compared this to the same skin areas of individuals without SD. By using exact amplicon sequence variants (ASVs) instead of operational taxonomic units (OTUs), we were able to zoom in at species level and made our results easier to compare [12].

## Materials and methods

### Study participants and sample collection

This is a cross-sectional study conducted within the Rotterdam Study. The Rotterdam Study started in 1990 and is an ongoing prospective population based cohort study in a middle aged and elderly population in the Ommoord district of Rotterdam, The Netherlands. All residents aged 45 years and above were invited to participate. At this point, the Rotterdam Study compromises over 15,000 subjects of predominantly North-European descent. Details of the study have been published before [13]. The Rotterdam Study has been approved by the Medical Ethics Committee of the Erasmus MC (registration number MEC 02.1015) and by the Dutch Ministry of Health, Welfare and Sport (Population Screening Act WBO, license number 1071272-159521-PG). The Rotterdam Study Personal Registration Data collection is filed with the Erasmus MC Data Protection Officer under registration number EMC1712001. The Rotterdam Study has been entered into the Netherlands National Trial Register (NTR; www.trialregister.nl) and into the WHO International Clinical Trials Registry Platform (ICTRP; www.who.int/ictrp/network/primary/en/) under shared catalogue number NTR6831. All participants provided written informed consent to participate in the study and to have their information obtained from treating physicians.

In 2016, collection of skin swabs of the nasolabial fold were introduced and are still ongoing. Participants were not allowed to use any skincare products on the day of the examination. Participants that did use skincare products were excluded (n = 108). Participants who used antibiotics in the past 6 months were also excluded (n = 126). Data collection for this research included participants with swabs until April 2018.

Seborrheic dermatitis was diagnosed by dermatology-trained physician during a full body skin examination. The SD diagnosis was based on a greasy scaling, erythema and a characteristic distribution in areas rich in sebaceous glands. Since the microbial swabs were taken from the nasolabial folds in the Rotterdam Study, participants with SD and involvement of the nasolabial fold were considered lesional cases. Participants with SD, but without involvement of the nasolabial fold were considered non-lesional cases. Participants in the Rotterdam Study without SD or any other skin diseases were considered as controls. No sample size calculation was performed.

### Swab collection, DNA extraction and 16S rRNA gene polymerase chain reaction amplification and sequencing

A cotton wool swab, pre-moistened with three drops of 0.9% NaCl fluid, was rubbed forth and back, in parallel position along the nasolabial fold (50 times, during 30 seconds). Samples were stored at -20°C for a maximum period of three hours and then at -140°C before processing. Negative swabs (air swabs) were taken for quality control of the sequencing.

DNA was extracted using the DNA Extraction Kit on the Arrow pipetting instrument (DiaSorin S.P.A., Saluggia, Italy). Swabs were treated with DNA Pre-treatment Buffer 2 and Proteinase K for 30 minutes at 56°C, followed by DNA isolation on the Arrow instrument according to the manufacturer’s protocol in batches of 12 samples per run. DNA concentration was measured using the Quant-iT PicoGreen dsDNA Assay Kit (Thermo Fisher Scientific, Waltham, MA) and DNA was stored at -20°C.

The V1 to V3 variable regions of the 16S rRNA gene were amplified using the 27F-519R primer pair and dual indexing as previously described [14, 15]. Amplicons were normalized and pooled in batches (total number of swabs per sequencing run: 300). The pools were purified using Agencourt AMPure XP (Beckman Coulter Life Science, Indianapolis, IN) and the quantity of the pool was assessed using the Quant-iT PicoGreen dsDNA Assay Kit. PhiX Control v3 library (Illumina Inc., San Diego, CA) was spiked into (~10%) the pool prior to sequencing on an Illumina MiSeq sequencer (MiSeq Reagent Kit v3, 2 x 300 bp). Samples were sequenced in three batches.

### Bioinformatic analysis

Raw reads from Illumina MiSeq were demultiplexed using a custom script to separate sample fastq files based on the dual index. Primers, barcodes and heterogeneity spacers were trimmed off using tagcleaner v0.16 [16]. Trimmed fastq files were further processed using the DADA2 pipeline [17]. In contrast to standard clustering of reads that cluster sequence reads based on a similarity threshold (usually 97%), and assign them to taxa to generate operational taxonomic units (OTUs), DADA2 allows to analyse exact amplicon sequence variants (ASVs) as the unit of analysis without imposing dissimilarity thresholds [17]. This is done by inferring the biological sequences before incorporating amplification and sequencing errors. Parameters used to run DADA2 are detailed in the S1 File.

### Data analysis

Demographic characteristics of participants included in the study were presented as proportions and compared between cases and controls using chi-squared statistics. We excluded ASVs belonging to contaminant phyla as detailed in Salter et al. [18]. Further, we included ASVs that were present in at least 5% of the total dataset and had a minimum of 1000 reads based on the percentile count distribution. Frequency plots of most abundant phyla in the dataset were calculated using relative abundances. Most abundant genera were plotted using different prevalence thresholds from (5 to 100%) using a heatmap plot.

To compare the profile of bacteria between SD patients against controls, we first calculated α-diversity per sample using Chao1 and Shannon diversity. Chao1 is an estimation of the richness of the sample (aka: how many ASV per group), while Shannon-diversity gives an estimate of the relative distribution of sequences among the ASVs. We tested for differences in Chao1 and Shannon diversity using Kruskal-Wallis tests and adjusted the p-values for pair-wise comparisons. We also calculated Shannon and Chao1 after rarefying all samples to have exactly 10000 counts. These analysis were done to evaluate whether any significant difference between the groups was due to different library sizes (details are presented in the S1 File).

Next, for downstream analysis we transformed the dataset using centered log-ratio approach to account for unequal library sizes. This data transformation is recommended to analyse data derived from Next Generation Sequencing since this data is assumed to be compositional (data conveys only relative information since the total abundance is unknown) [19, 20]. Because the logarithmic normalization does not handle zeros, these needed to be imputed first. The imputation of zeroes can be done under different assumptions (see Palarea-Albaladejo et al. for details [21]). Here, we assumed that any feature (ASV) observed in more than one sample could appear in another sample if sequenced with infinite depth, which can be modelled using Bayesian-Multiplicative replacement of count zeros [21].

To visualize whether the microbiome composition clustered per sample characteristics (here, being a case or control, or other sample characteristics), we used Principal Component Analysis (PCA). Further, we tested for differences in the microbiome composition using permutational multivariate analysis of variance (permANOVA) [22] and adjusting for sex, age (as a continuous variable) and batch variables.

Finally, we performed compositional data analysis to test for differential abundance between specific ASVs for seborrheic (lesional and non-lesional) cases and controls with lesional cases used as the reference groups using the ALDEx2 package [23]. The package uses centered log-ratio transformation of the data to correct for unequal library sizes. We tested for differential composition using a generalized model, while adjusting for sex, age and batch as covariates. Significant p-values were defined with a threshold of p-value = 0.05 and borderline with a p-value of 0.1. Of note, the package considers significant effects when the absolute value of the coefficients are higher than 1. However we considered estimates significant if the p-value was <0.05 after multiple testing (Benjamini-Hochberg Procedure, adjusted p-values are given by the package).

For the analysis of alpha and beta diversities the phyloseq and microbiome packages were used [24] (https://github.com/microbiome/microbiome). The pairwise comparison and graphical display of the alpha-diversity was performed using library ("ggstatsplot") retrieved from https://cran.r-project.org/web/packages/ggstatsplot/index.html.

PermANOVA analysis was done using the function “adonis” from the vegan package (https://cran.r-project.org/web/packages/vegan/index.html). Imputation of zeroes was done using the Z compositions packages. The generation of plots and statistical analysis were done using phyloseq and microbiome packages. All the packages were implemented in Rv.3.5.2 (Eggshell Igloo version). Functions and parameters used for these analysis are detailed in the S1 File.

### Phylogenetic analysis

To improve the taxonomic resolution of the ASV that showed differential composition between lesional cases and controls, an exploratory phylogenetic analysis was done. We performed this by aligning the sequence reads of the ASVs that showed differential composition between cases and controls with the partial 16S rRNA sequences from different *Staphylococcus s*pecies retrieved from GenBank (reference numbers were obtained from Ghebremedhin B. et al.) [25]. After the multiple sequencing alignment, a phylogenetic tree was constructed using the neighbour-joining algorithm. The phylogenetic analysis was implemented in the package MEGA [26]. Details on the programs and parameters for generation of the phylogenetic tree are presented in the S1 File.

## Results

Skin swabs were collected from 562 participants. Of these 75 participants were classified as non-lesional cases and 22 as lesional cases. Demographic characteristics are shown in Table 1. Most of the participants were male in both non-lesional and lesional cases. The median age was 53 years (inter-quantile range; IQR: 48–64 years) in the control group, and 56 years (IQR: 49–55 years) for non lesional cases and 68 years (IQR: 49–55 years) for lesional cases. The quantile age distribution was significantly different with lesional cases being older than the controls.

**Table 1:** Descriptive statistics of participants with skin swabs.

		Controls (n = 465)	Non-lesional Cases (n = 75)	lesional Cases (n = 22)	P-value*
**Sex**					<0.00004
	Men	210 (77%)	44 (16%)	20 (7%)	
	Women	255 (89%)	31 (11%)	2 (0.7%)	
**Batch**					0.042
	1	215 (46%)	46 (61%)	9 (41%)	
	2	250 (54%)	29 (39%)	13 (59%)	
**Age quantiles (years)**					0.01
	40–49	130 (28%)	18 (24%)	1 (5%)	
	50–54	113 (24%)	13 (17%)	5 (23%)	
	55–65	116 (25%)	20 (27%)	2 (9%)	
	> 65	106 (23%)	24 (32%)	14 (64%)	

* Chi-square test of the comparisons between lesional non lesional and controls.

### Bacterial community profiles of the naso-labial fold

In total, 15651 amplicon sequencing variants (ASVs) were identified in 562 individuals. The ASVs were assigned to 27 unique phyla (S1 Fig), some of which were clearly contaminant phyla including *Planctomycetes* and *Cyanobacteria*. The total number of reads was 12277291 with a median count of 21340 (range: 0–218661) per sample. After quality control that included the filtering of contaminant ASVs and samples with low counts and outliers (S1 File), and including ASVs present in at least 5% of the total dataset, 185 ASVs in 529 samples (controls: 436; non-lesional cases:71; lesional cases:22) remained.

**Fig 1:**
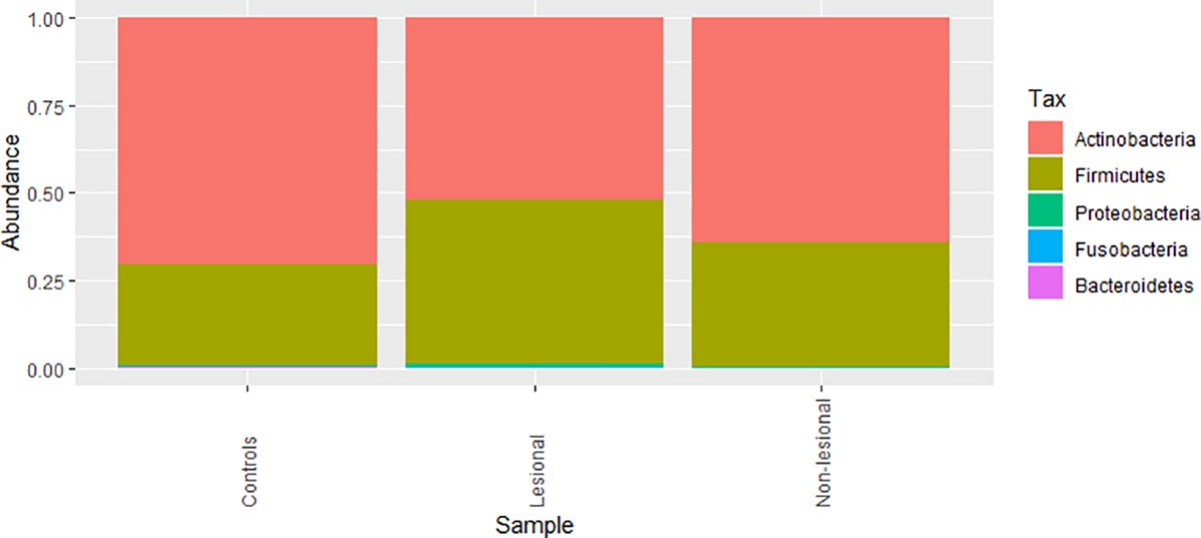
Barplot of relative frequency of most common phyla averaged per control, non-lesional and lesional cases.

Fig 1 presents the relative frequency of phyla in the filtered dataset averaged per control SD cases groups. As shown in Fig 1, we identified five major phyla in the data set, namely: *Actinobacteria*, *Proteobacteria*, *Bacteroidetes*, *Firmicutes* and *Fusobacteria*.

**Fig 2:**
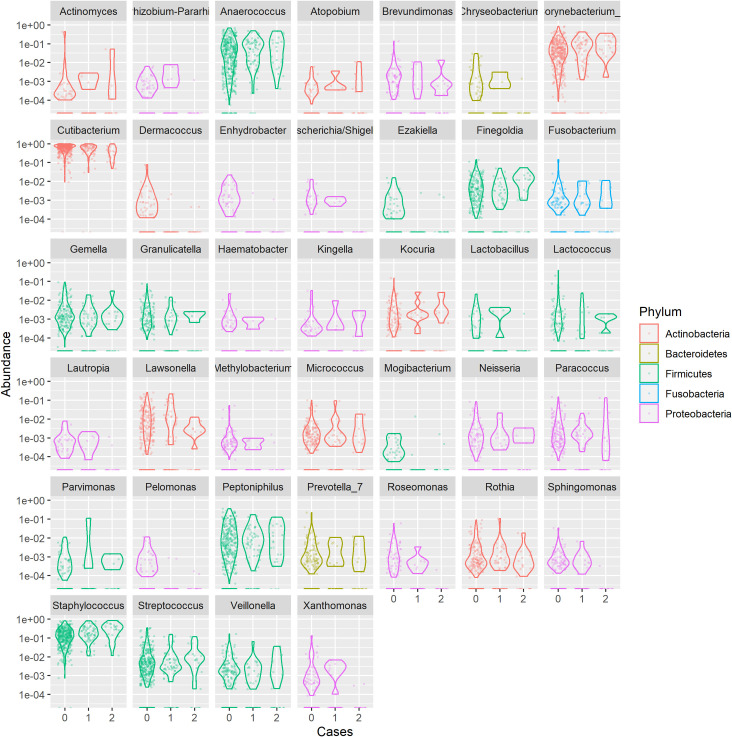
Barplot of relative frequency of genera per phylum. The relative abundance of main genera is presented separately, with controls = 0, non-lesional cases = 1 and lesional cases = 2. Only *Corynebacterium*_*1* was nominally significantly different between lesional SD versus controls.

There was an apparent increase in the relative abundance of *Firmicutes* in lesional cases when compared with controls at phylum level as seen in the plot. However, testing the differential composition of SD lesional, non lesional and controls was not significant. Fig 2 showed that several genera seemed to be increased in the lesional cases. However, after testing, only *Corynebacterium_1* was nominally significant (p-value< = 0.05; Aldext text; S2 Table).

**Fig 3:**
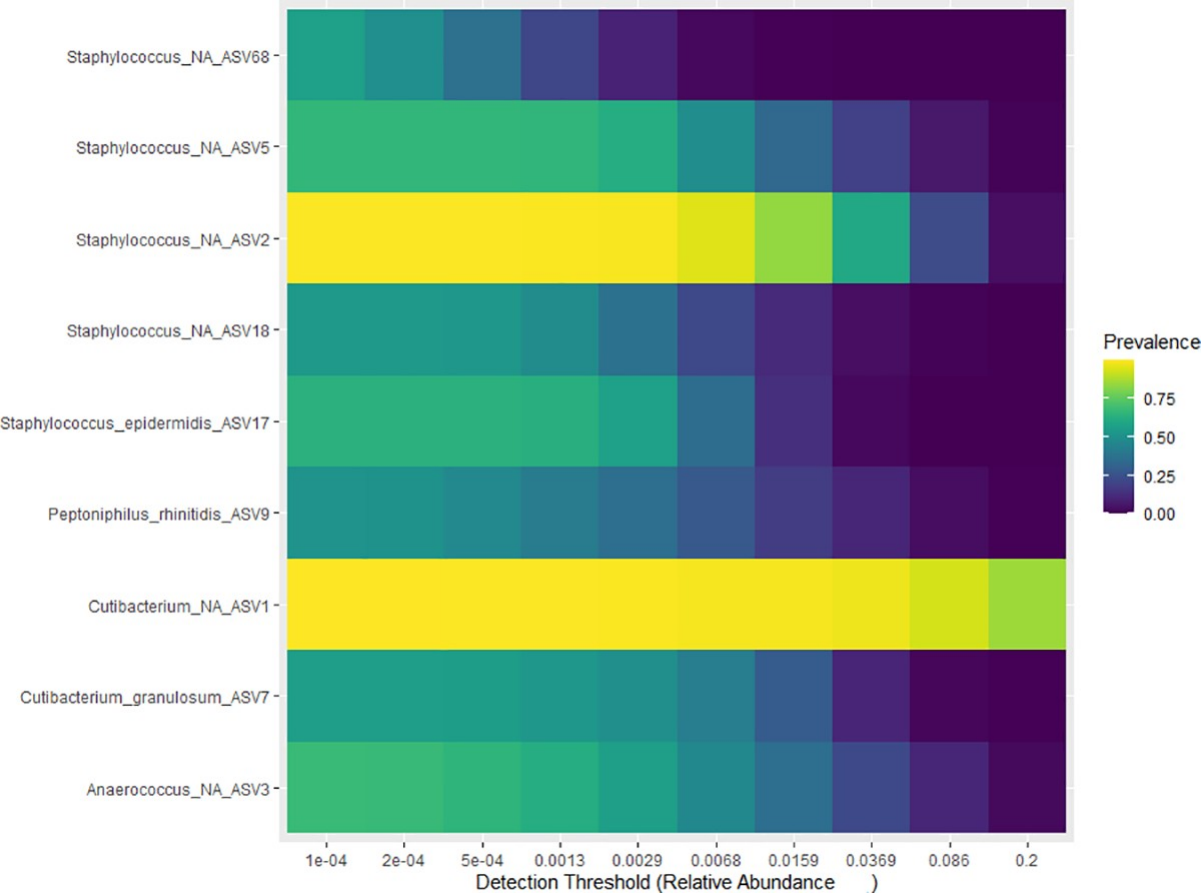
Heatmap plot of the most prevalent ASV identified in the sample. Fig 3 presents the most prevalent ASVs (y-axis) identified at different detection thresholds (0.001 to 20%). Most common ASVs appear at low abundance per sample (0.01 to 1%).

Fig 3 presents a heatmap of ASVs identified at different prevalence estimates in all samples (*y-axis*) and at different levels of detections per sample; from 0.0001% to 20% (*x-axis*). As shown in this plot, one member of the genus *Cutibacterium* (ASV1) was the most prevalent and was found in over 75% of all samples with relative abundances of 3% and higher. The second most prevalent ASV belonged to the genus *Staphylococcus* (ASV2) with a prevalence of 75% at lower abundances (< = 2%). Other ASVs present in at least 50% of the samples included other ASVs belonging to *Staphylococcus* species *(*ASV68, ASV5, ASV18; *Staphylococcus epidermidis*), *Cutibacterium granulosum* (ASV7) *Peptoniphilus rhinitidis* and *Anaerococcus*.

To investigate whether the participants clustered based on their disease status we used PCA. S2 Fig displays the first two axis of variation in the cutaneous microbiome composition. The first component explained 13% of the variation of the data and the second component explained 7%. However, there was not a clear separation between cases and controls.

### Comparison of the microbiome composition between SD and controls

**Fig 4:**
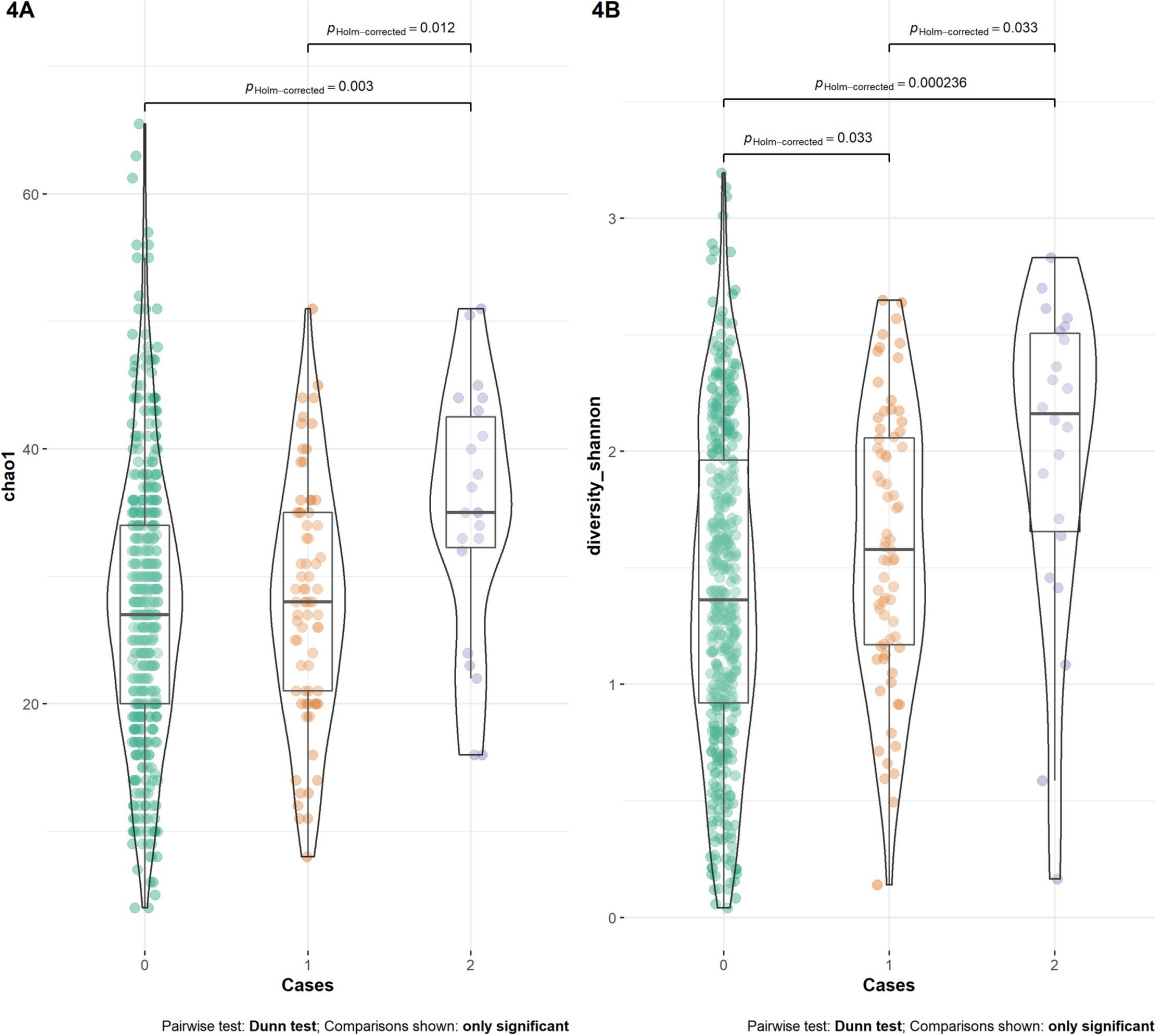
Boxplot of α-diversity. Fig 4A presents the violin-plot of Chao1 diversity in controls, lesional and non-lesional cases. Fig 4B presents the violin-plot of Shannon diversity in controls, lesional and non-lesional cases. P-values are adjusted pair-wise comparisons. Overall Kruskal-Wallis test is described in the text.

We estimated α-diversity of our data per group (controls; non-lesional and lesional SD) using Shannon-diversity and Chao1 statistics. As shown in Fig 4A, the median Chao1 was significantly higher for lesional SD cases in comparison with both non-lesional cases and controls (Kruskal-Wallis rank sum: Chi-squared: 11.14; global p-value = 0.004; controls vs lesional cases; adjusted p-value = 0.003; lesional cases vs non-lesional cases; adjusted p-value = 0.0012). Shannon α-diversity estimates were also significantly different between lesional cases and controls (Kruskal-Wallis rank sum: Chi-squared: 19.06; global p-value = 7.3x10^-5^; lesional cases vs controls; adjusted p-value = 2.4x10^-4^; lesional cases vs non-lesional cases; adjusted p-value = 0.03; non-lesional cases versus controls: adjusted p-value = 0.03). Since alpha diversity parameters are sensitive to differences in sample library size, we evaluated the impact of different filtering thresholds as well as rarefaction (S1 File). As shown in the S3–S5 Figs, alpha diversity estimates were significantly higher in lesional cases when compared with non-lesional cases and controls at different filtering thresholds and after rarefying (S1 File).

#### Multivariate analysis

We tested for differences in the microbiome composition between SD cases and controls using permANOVA. We found that overall, the composition of the microbiome differed between cases and controls (R^2^ = 0.1%; p-value = 0.03). In addition, age, sex and batch effects were also significant (age: p-value = 0.009; R^2^ = 0.6%; sex: p-value: 001; R^2^ = 2%; batch: p-value = 0.001; R^2^ = 1%).

#### Univariate analysis

We investigated whether there was a difference in the composition of ASVs between SD and controls adjusting for known sex, age and batch variables. We found nominally significant differences in the composition between lesional cases compared against controls for bacteria of the genus *Staphylococcus* including ASV18 (effect size -3.37; p-value = 0.01), ASV15 (effect size: -2.97; p-value = 0.02); ASV22 (effect size -2.88; p-value = 0.03), ASV13 (effect size -2.64; p-value = 0.04) and ASV5 (effect size -2.41;p-value = 0.05). Other bacteria with differences between lesional and controls included a member of the genus *Micrococcus* (ASV137), *Finegoldia* (ASV60), and *Lawsonell*a (ASV32). None of the differences were significant after correcting for multiple testing (Table 2). *Cutibacterium* (ASV1), which was most prevalent in this cohort, and nominally significant at genus level was only borderline associated with SD (effect size:-0.83, p-value: 0.07).

**Table 2:** Association analysis between ASV composition (ASV individual level) and seborrheic dermatitis (with categories controls, non-lesional cases and lesional cases).

Bacteria	Effect size^a^	P-value^a^	Effect size^b^	P-value^b^	Genus	Species
ASV18	**-3.37 (1.16)**	**0.01**	-2.22 (1.28)	0.09	Staphylococcus	NA
ASV15	**-2.97 (1.23)**	**0.02**	-2.35 (1.35)	0.1	Staphylococcus	NA
ASV22	**-2.88 (1.21)**	**0.03**	-2.39 (1.33)	0.09	Staphylococcus	NA
ASV13	**-2.64 (1.22)**	**0.04**	-1.39 (1.34)	0.32	Staphylococcus	NA
ASV137	**-2.13 (0.91)**	**0.04**	**-2.33 (1.01)**	**0.05**	Micrococcus	NA
ASV5	**-2.41 (1.19)**	**0.05**	-0.88 (1.31)	0.51	Staphylococcus	NA
ASV20	2.43 (1.15)	0.06	1.5 (1.3)	0.3	Anaerococcus	NA
ASV68	-1.54 (0.81)	0.07	-1.18 (0.9)	0.21	Staphylococcus	NA
ASV63	1.89 (0.98)	0.1	2.0 (1.1)	0.13	Staphylococcus	NA
ASV60	-1.89 (0.96)	0.11	-2.23 (1.06)	0.07	Finegoldia	NA
ASV171	-1.67 (0.89)	0.11	1.90 (0.1)	0.11	Streptococcus	sanguinis
ASV25	-1.83 (1.08)	0.12	-1.06 (1.20)	0.4	Anaerococcus	NA
ASV32	1.91 (1.08)	0.13	1.30 (1.20)	0.37	Lawsonella	NA

^a^ Effect sizes and p-values of the comparison controls versus lesional cases (with negative effects meaning ’relative decrease’ in controls relative to lesional cases.

^b^ Effect sizes and p-values of the comparison non-lesional versus lesional cases (with negative effects meaning ’relative decrease’ in non-lesional cases relative to lesional cases. Effect sizes larger than 1 are considered significant [20]. Analysis were adjusted for age, sex and Batch effects.

### Phylogenetic analysis of ASV with suggestive differential composition between lesional SD and controls

**Fig 5:**
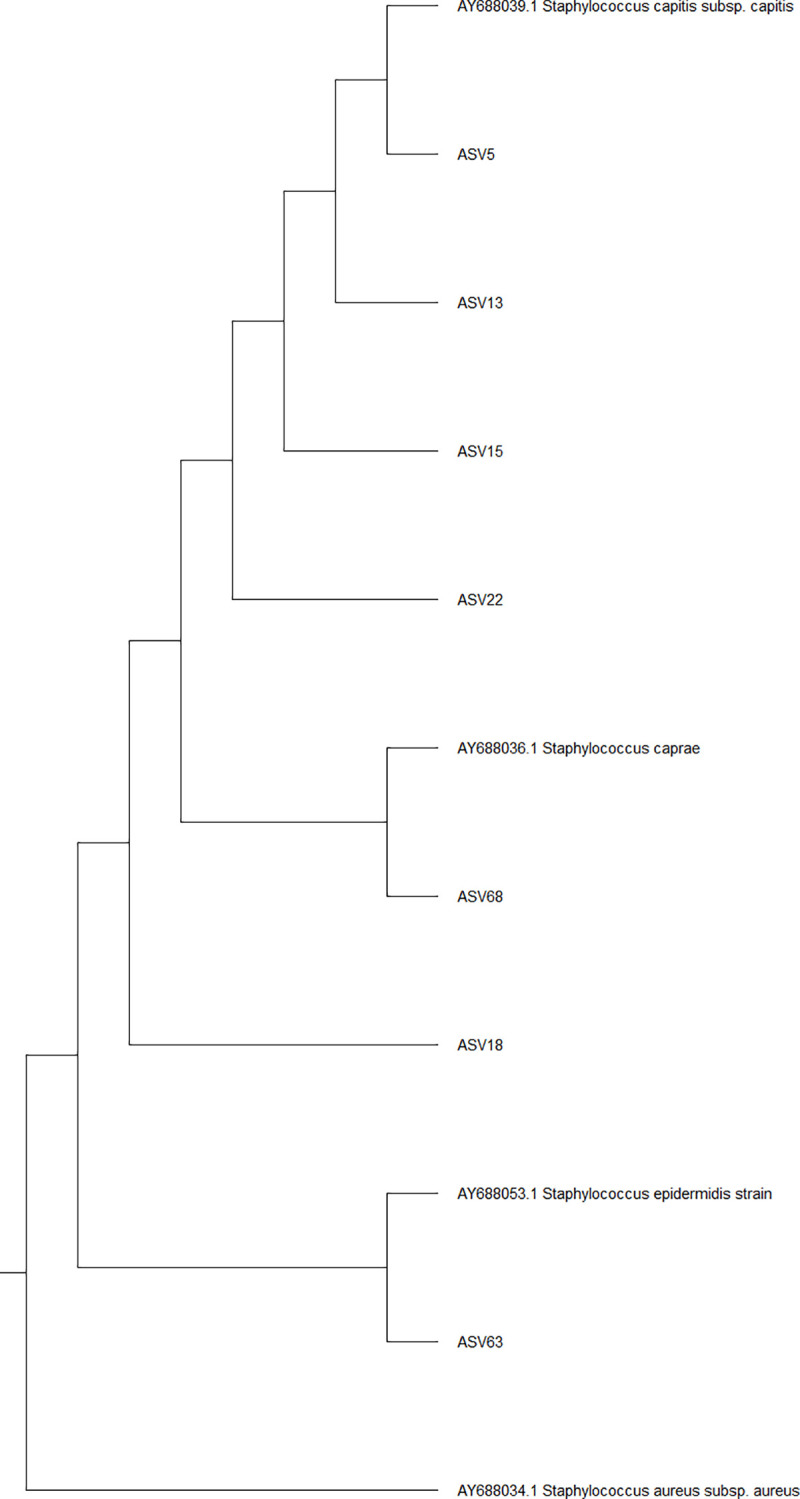
Phylogenetic tree of the ASVs with suggestive differences in composition between lesional SD and controls. Fig 5 depicts the phylogenetic relationship between ASVs assigned to *Staphylococcus* species and *Staphylococcus* species obtained from GenBank (16S rRNA sequence). This sequences begin with AY*. ASVs closely related to *S*. *capitis* belong to the same node (ASV5, ASV13, ASV15 and ASV22). ASV68 appears to belong to *S*. *caprae* together with ASV18. ASV63 clustered with *S*. *epidermidis*. Sequence of *S*. *aureus* appear as outgroup (sequence with most differences).

As shown in the Table 2, seven out of 12 ASVs that were (nominally) significantly different between controls and lesional cases were members of the genus *Staphylococcus*, most of which showed increased composition in lesional cases in comparison with controls. Of these, none of them could be annotated to species level by the DADA2 pipeline. To better understand the role of this genus in SD, we compared the DNA sequence of the reads with the 16S rRNA sequences available from different strains of Staphylococci available from GenBank (Fig 5). As shown in this figure, the ASVs with differences in composition between lesional cases and controls were closely related to *S*. *capitis* (ASV5, ASV13, ASV15; ASV22) *S*. *caprae* (ASV) and *S*. e*pidermidis* (ASV33) and were separate from the from *S*. *aureus* sequence.

## Discussion

In this large cross-sectional study comparing the cutaneous microbiome of individuals with SD with individuals with no skin disease (controls), we found a significant increase in the α-diversity between lesional cases and controls. Comparing the overall microbiome composition between cases and controls using a multivariate approach also led to significant differences between the groups, although the contribution of skin condition was small in comparison to other sample characteristics such as sex. In the design of the study we assumed that the bacterial microbiome of non-lesional skin would also differ from healthy participants as Soares et al. showed this for people with dandruff [27], but we did not find a clear separation between non-lesional cases and controls. In the univariate analysis we found differences in the composition of members of specific bacteria from the genus *Staphylococcus*, thought to be members of the normal skin flora, including: *S*. *capitis*, *S*. *caprae* and *S*. *epidermidis*, with most of them being increased in lesional cases compared to controls. No differences were found for *S*. *aureus* in this analysis. Other bacteria with an increased abundance in the cases were members of the genus *Micrococcus*, *Finegoldia* sp. and *Streptococcus sanguinis*. The changes found in the univariate analysis were not statistically significant after correcting for multiple testing. Though, especially for the *Staphylococcus* genus there seems to be a trend, as seen in the comparison between non-lesional and lesional SD groups (Table 2). The principal component analysis did not show a clear separation of microbiome composition between controls and cases. In the previous smaller study by Tanaka et al., a separation between cases and control was suggested, although their graph leaves room for discussion and no statistical test was performed [28]. We replicated the findings that *Staphylococcus* and *Streptococcus* have an increase abundance in lesional skin.

In our study we found similar distribution of bacteria from sebum rich areas in our dataset as in previous studies [29]. Using a prevalence of 50% to define a ’core’ microbiome, we found that members of *Cutibacterium* species (ASV1_ *Cutibacterium _NA* and *Cutinebacterium granulossum*) and *Staphylococcus* species (*Staphylococcus epidermidis* and *Staphylococcus _NA*) were the most abundant, which serves as an internal validation of our dataset. Of this ‘core’ bacteria only one member of the *Cutibacterium* species seemed to have differences in their ‘abundance’ between SD cases and controls as shown in the univariate analysis (Table 2). The other ASVs with suggestive differences in the abundance between cases and controls were not amongst the core set, which suggests that the most differences between lesional seborrheic dermatitis and controls occur for bacteria that have relatively low (relative) abundances between 5% to 10%.

We found differences in the relative composition of members of the *Staphylococcus* genus, which supports previous findings of a differential composition of this genus in seborrheic dermatitis based on smaller series of cases [11]. *Staphylococcus* species include different species known to be part of normal skin flora, such as *S*. *epidermidis*, *S*. *capitis* and *S*. *caprae*, as well as pathogens such as *S*. *aureus*. Because in most studies on seborrheic dermatitis the analysis of bacterial microbiome is done at genus level, it is difficult to compare our results with these from previous studies since we chose to analyse exact ASV instead of grouping ASVs into genera. In a recent study an increased abundance of *S*. *epidermidis* in patients with impaired skin barrier was found [30]. Because commensal bacteria can also act as opportunistic pathogens under special circumstances (e.g.: impaired barrier function), the previous study as well as our results supports the hypothesis that commensal bacteria rather than pathogens may be associated with SD.

The higher alpha diversity in SD cases (both lesional and non lesional) in comparison to the controls is in contrast with studies on bacterial microbiome in other chronic inflammatory skin conditions such as atopic dermatitis, where a decreased alpha diversity in lesional skin has been observed. Although both diseases are associated with a skin barrier impairment, it is likely that the role of microbial dysbiosis is different for the two skin conditions. In atopic dermatitis, alpha diversity is lower due to the predominance of one bacterium, namely: *S*. *aureus*, which is considered more of a pathogen than a commensal [31]. Higher *S*. *aureus* colonization has been associated with immune dysfunction and a decrease of the production of antimicrobial peptides, which will favour the overgrowth of this bacteria while inhibiting the growth of competing bacteria [32]. We looked at the total number of 47 ASVs related to *Staphylococcus* species in our data and did a phylogenetic analysis with all sequences available at GenBank for 16S rRNA for a total of 75 sequences. Interestingly, only one of 47 ASVs in our data was phylogenetically closer to *S*. *aureus* than to the rest (ASV109: S6 Fig), which suggests that *S*. *aureus* is not a major player in SD. It could also be argued that an affected nasolabial skin could be a niche for bacteria from nearby areas such as mouth (e.g. *Streptococcus sanguinis*) or nose (*Peptonilus rinitidis*). ASVs associated with these bacteria seemed to be increased in SD (when compared with controls), although the differences were not statistically significant. This also may explain the increase in the diversity seen in our dataset. Thus, one could argue that in SD subjects the increased diversity is secondary to an impaired skin barrier, which leads to the colonization of otherwise commensal bacteria.

As mentioned above, we chose exact ASVs as the unit of analysis instead of OTUs in an attempt to gain taxonomic resolution beyond genus level, since the pipeline can annotate 1 to 2 nucleotides difference between ASVs. However, because the DADA2 bioinformatic pipeline uses a strict threshold to annotate ASVs to bacteria from reference databases such as SILVA (100% match; in other words identical sequences), many of the ASVs could not be annotated to species level, and were left as ‘unknown’ species. In most studies, this issue is solved by grouping ASVs or OTUs into genera as unit of analysis, which may lead to spurious results, because different species with different roles in skin biology or pathology (*e*.*g*.: *S*. *aureus* versus *S*. *epidermidis*) are then assumed to be the same. With the methods we used, we could have agglomerated similar ASVs into species level (*e*.*g*. different ASVs associated with *S*. *epidermidis* into one single *S*. *epidermidis*), but due to the high proportion of ASVs that were unknown at species level, we would not have enough data to analyse. With this, we showed that trying to separate species from the same genus may be valuable to understand the role of specific bacteria in SD. Because whole genome sequencing is still expensive for large studies, especially when analysing microbiome from low to medium biomass, a better annotation of reference datasets and perhaps sequencing of another region or even the whole gene could improve the resolution in future studies.

Our study has some limitations. First, this study includes a middle aged and elderly population, and as the microbiome composition changes with age, this might limit the generalizability to younger patients. Second, the bacterial biomass of the skin is lower than that from other human niches [33]. This has implications for sequencing since samples with low to medium levels of extracted DNA are more prone to contamination by other bacteria, which can lead to spurious results. Here we excluded obvious contaminant phyla based on previous publications and filtered ASVs based on prevalence. Third, although our sample of cases and controls is much larger than most studies on skin microbiome, the number of lesional cases was probably too low in comparison with the number of controls, which had much larger variation and made it difficult to separate cases from controls in the PCA. The low density of skin bacterial DNA also creates problems in the statistical analysis since the data were highly sparse. We used methods that alleviated this issue (imputing zeroes), but still the analysis may have been too conservative to survive multiple testing. Further, most methods assume that rare species are not important in determining variation but this may not be true as shown here in our study. To alleviate this, larger sample sizes will be needed to confirm our results.

To conclude, we found differences in the microbiome composition in a large sample of patients with SD when compared with controls. There was a trend towards higher prevalence of members of the genus *Staphylococcus*, most of which showed increased composition in lesional cases, and were closely related to *S*. *capitis S*. *caprae* and *S*. e*pidermidis*. The associations were not significant when correcting for multiple testing. Future studies are required to validate these findings.

## Supporting information

S1 Fig Frequency of 27 unique phyla presented. The graphs present the taxa prevalence against total counts, with each dot representing a different taxa.
(TIF)

S2 Fig Principal component analysis (PCA) of microbiome composition (the centered log- ratios). No clear separation of microbiome composition was observed between controls (grey dots) and cases separated in non-lesional (blue dots) and lesional SD cases (green dots)
(TIF)

S3 Fig Boxplot of α-diversity with minimal filtering. S3A Fig presents the violin-plot of Chao1 diversity in controls, lesional and non-lesional cases. S3B Fig presents the violin-plot of Shannon diversity in controls, lesional and non-lesional cases. P-values are adjusted pair-wise comparisons.
(TIF)

S4 Fig Boxplot of α-diversity before 5% prevalence filtering. S4A Fig presents the violin-plot distribution of Chao1 diversity in controls, lesional and non-lesional cases. S4B Fig presents the violin-plot of Shannon diversity in controls, lesional and non-lesional cases. P-values are adjusted pair-wise comparisons.
(TIF)

S5 Fig Boxplot of α-diversity after rarefying. S5A Fig presents the violin-plot of Chao1 diversity in controls, lesional and non-lesional cases. S5B Fig presents the violin-plot of Shannon diversity in controls, lesional and non-lesional cases. P-values are adjusted pair-wise comparisons.
(TIF)

S6 Fig Phylogenetic tree of the ASVs belonging to the genus *Staphylococcus*. The figure depicts the phylogenetic relationship between ASVs assigned to *Staphylococcus* species and *Staphylococcus* species obtained from GenBank (16S rRNA sequence). This sequences begin with AY*. Only ASV109 was phylogenetically closer to *S*. *aureus*.
(TIF)

S1 Table Association analysis between microbiome composition (phylum level) and seborrheic dermatitis (with categories controls, non-lesional cases and lesional cases. ^a^ Effect sizes and p-values of the comparison: controls versus lesional cases. ^b^ Effect sizes and p-values of the comparison: non-lesional cases versus lesional cases.
(DOCX)

S2 Table Association analysis between microbiome composition (genus level) with seborrheic dermatitis (with categories controls, non-lesional and lesional cases) and with lesional cases as reference groups. ^a^ Effect sizes and p-values of the comparison: controls versus lesional cases. ^b^ Effect sizes and p-values of the comparison: non-lesional cases versus lesional cases.
(DOCX)

S1 File (DOCX)

## Abbreviations

SD: Seborrheic dermatitis
OTUs: operational taxonomic units
PCA: principal component analysis
ASVs: amplicon sequencing variants
