# Supplementary figures and images for: Composition of cutaneous bacterial microbiome in seborrheic dermatitis patients: A cross-sectional study
Source: PLoS One. 2021 May 24;16(5):e0251136. doi: 10.1371/journal.pone.0251136 (PMC8143393; doi:10.1371/journal.pone.0251136)

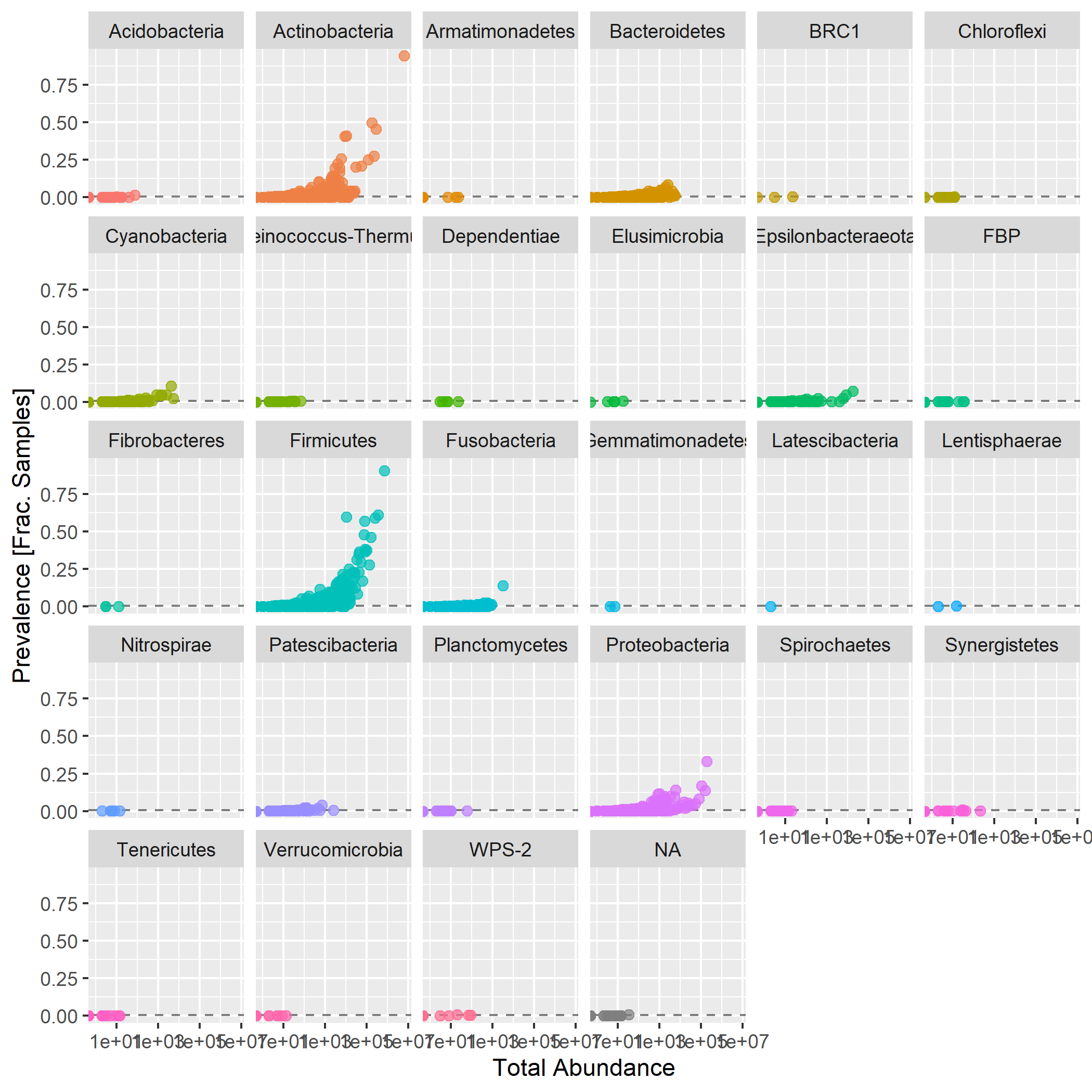

Supplement: S1 Fig — The graphs present the taxa prevalence against total counts, with each dot representing a different taxa. (TIF) [file pone.0251136.s001.tif]

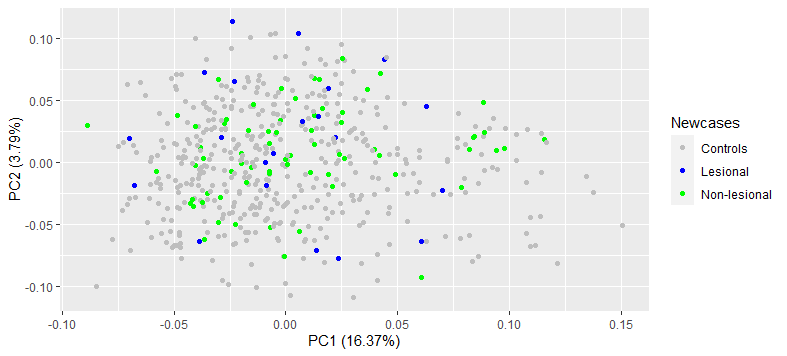

Supplement: S2 Fig — No clear separation of microbiome composition was observed between controls (grey dots) and cases separated in non-lesional (blue dots) and lesional SD cases (green dots) (TIF) [file pone.0251136.s002.tif]

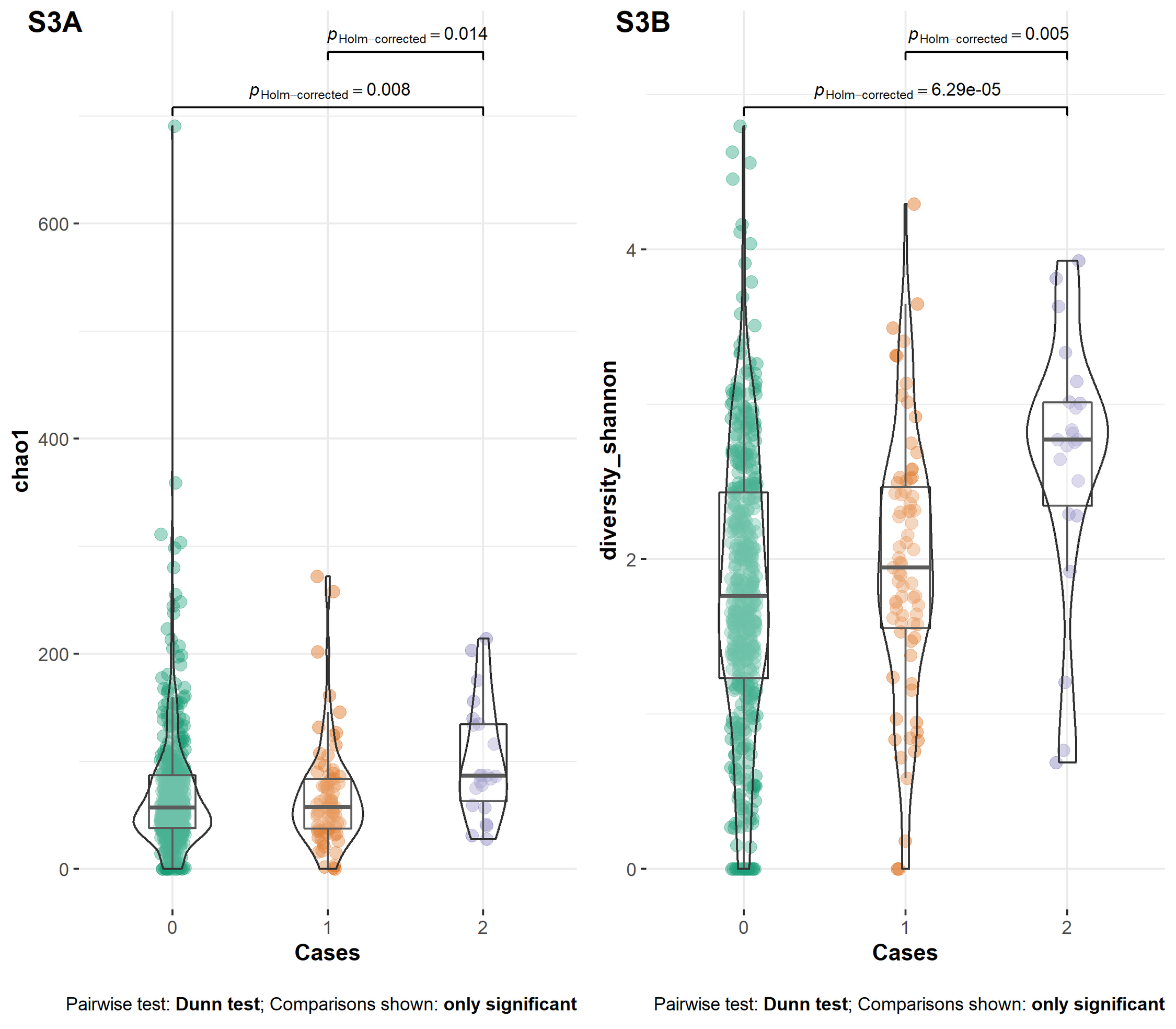

Supplement: S3 Fig — S3A Fig presents the violin-plot of Chao1 diversity in controls, lesional and non-lesional cases. S3B Fig presents the violin-plot of Shannon diversity in controls, lesional and non-lesional cases. P-values are adjusted pair-wise comparisons. (TIF) [file pone.0251136.s003.tif]

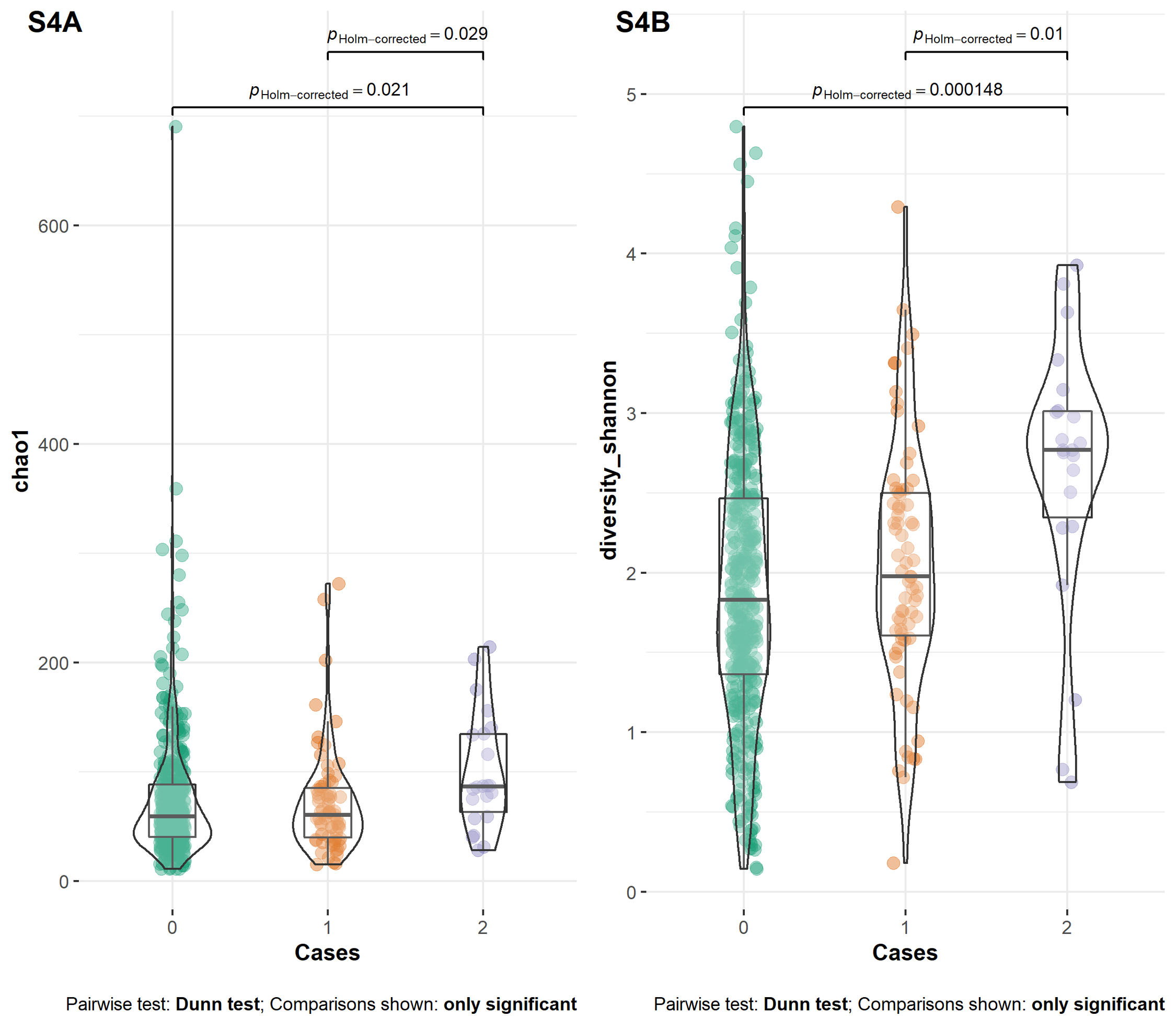

Supplement: S4 Fig — S4A Fig presents the violin-plot distribution of Chao1 diversity in controls, lesional and non-lesional cases. S4B Fig presents the violin-plot of Shannon diversity in controls, lesional and non-lesional cases. P-values are adjusted pair-wise comparisons. (TIF) [file pone.0251136.s004.tif]

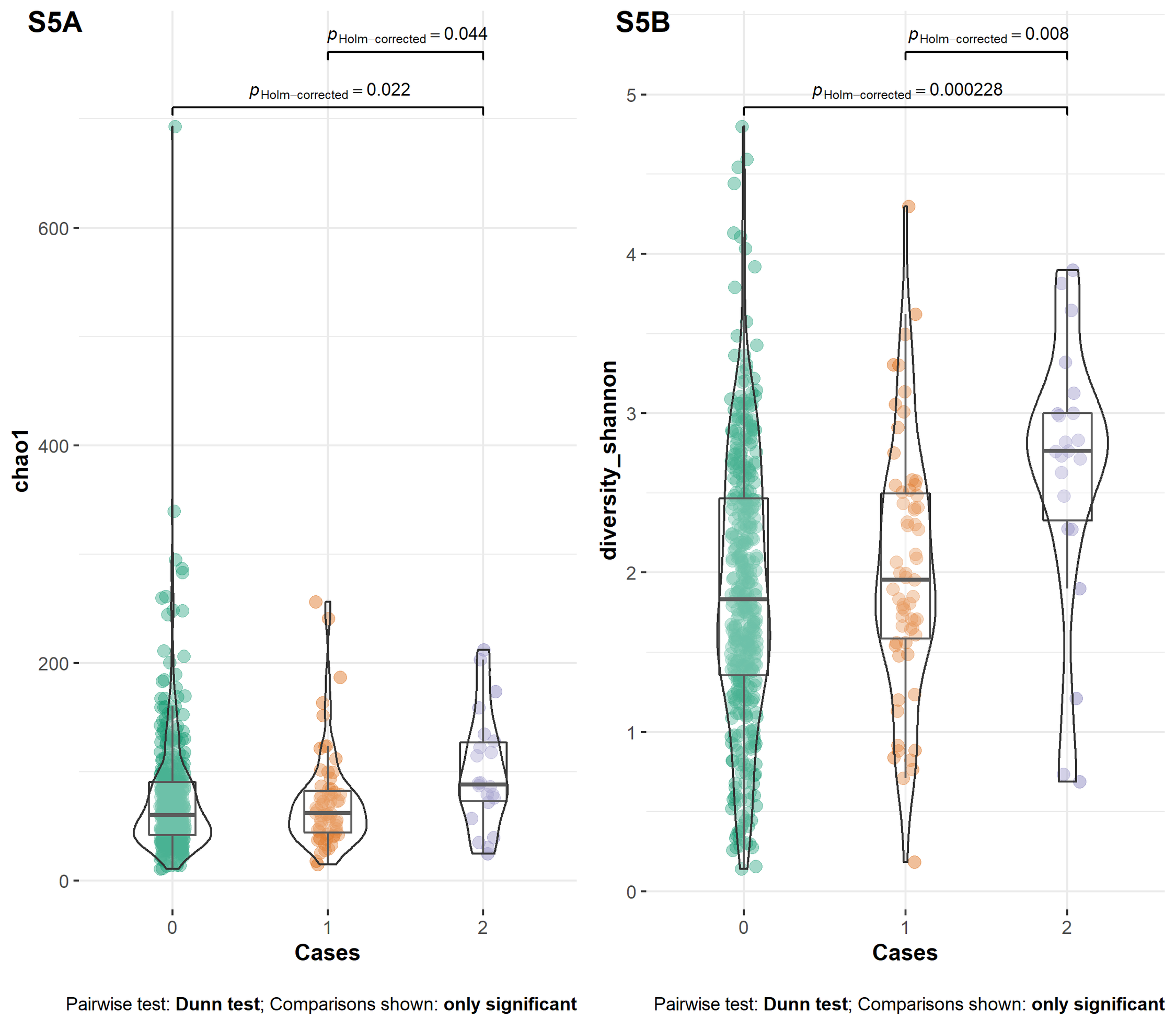

Supplement: S5 Fig — S5A Fig presents the violin-plot of Chao1 diversity in controls, lesional and non-lesional cases. S5B Fig presents the violin-plot of Shannon diversity in controls, lesional and non-lesional cases. P-values are adjusted pair-wise comparisons. (TIF) [file pone.0251136.s005.tif]

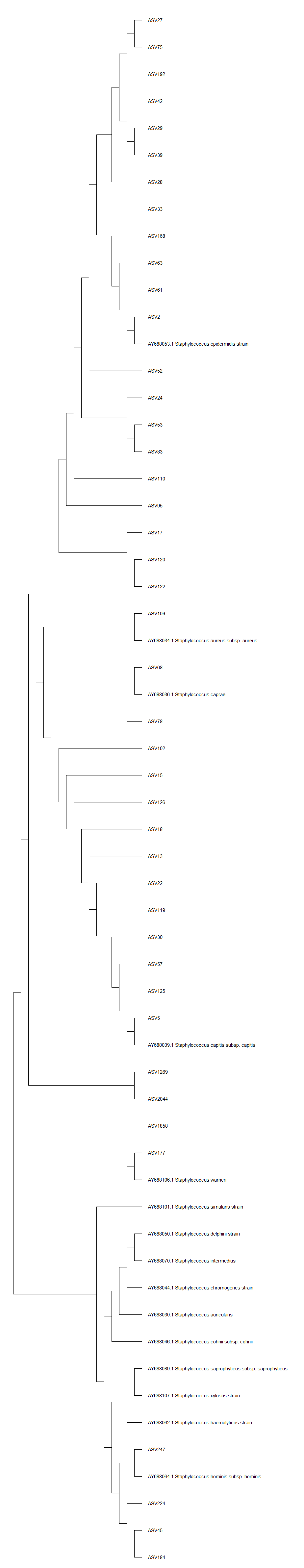

Supplement: S6 Fig — The figure depicts the phylogenetic relationship between ASVs assigned to Staphylococcus species and Staphylococcus species obtained from GenBank (16S rRNA sequence). This sequences begin with AY*. Only ASV109 was phylogenetically closer to S. aureus. (TIF) [file pone.0251136.s006.tif]
