# Supplementary material for: Composition of cutaneous bacterial microbiome in seborrheic dermatitis patients: A cross-sectional study
Source: PLoS One. 2021 May 24;16(5):e0251136. doi: 10.1371/journal.pone.0251136 (PMC8143393; doi:10.1371/journal.pone.0251136)
Supplement: S1 Table — a Effect sizes and p-values of the comparison: controls versus lesional cases. b Effect sizes and p-values of the comparison: non-lesional cases versus lesional cases. (DOCX) [file pone.0251136.s007.docx]

**S1 Table. Association analysis between microbiome composition (phylum level) and seborrheic dermatitis (with categories controls, non-lesional cases and lesional cases**

| **Bacteria** | **Effect size^a^** | **P-value^a^** | **Effect size^b^** | **P-value^b^** |
| --- | --- | --- | --- | --- |
| Actinobacteria | -0.24 (0.26) | 0.39 | -0.76 (0.45) | 0.12 |
| Firmicutes | 0.45 (0.27) | 0.12 | 0.65 (0.46) | 0.19 |
| Proteobacteria | -0.15 (0.44) | 0.67 | 0.04 (0.76) | 0.75 |
| Fusobacteria | 0.50 (0.42) | 0.30 | 0.31 (0.72) | 0.53 |
| Bacteroidetes | -0.57 (0.44) | 0.28 | -0.25 (0.75) | 0.60 |

^a^ Effect sizes and p-values of the comparison: controls versus lesional cases. ^b^ Effect sizes and p-values of the comparison: non-lesional cases versus lesional cases.
