# Supplementary material for: Composition of cutaneous bacterial microbiome in seborrheic dermatitis patients: A cross-sectional study
Source: PLoS One. 2021 May 24;16(5):e0251136. doi: 10.1371/journal.pone.0251136 (PMC8143393; doi:10.1371/journal.pone.0251136)
Supplement: S2 Table — a Effect sizes and p-values of the comparison: controls versus lesional cases. b Effect sizes and p-values of the comparison: non-lesional cases versus lesional cases. (DOCX) [file pone.0251136.s008.docx]

**S2 Table. Association analysis between microbiome composition (genus level) with seborrheic dermatitis (with categories controls, non-lesional and lesional cases) and with lesional cases as reference groups**

| **Genus** | **ASV_name** | **Effect size^a^** | **P-value^a^** | **Effect size^b^** | **P-value^b^** |
| --- | --- | --- | --- | --- | --- |
| Cutibacterium | ASV1 | 1.001 (0.468) | 0.04 | 0.371 (0.522) | 0.49 |
| Staphylococcus | ASV2 | -0.797 (0.417) | 0.06 | -0.312 (0.466) | 0.51 |
| Peptoniphilus | ASV9 | -1.849 (1.149) | 0.12 | -1.446 (1.284) | 0.28 |
| Anaerococcus | ASV3 | -1.403 (0.945) | 0.14 | -0.435 (1.055) | 0.68 |
| Finegoldia | ASV31 | -1.549 (1.061) | 0.18 | -1.527 (1.185) | 0.24 |
| Sphingomonas | ASV390 | 1.246 (0.812) | 0.22 | 1.027 (0.906) | 0.33 |
| Micrococcus | ASV137 | -1.245 (0.929) | 0.23 | -1.727 (1.037) | 0.15 |
| Granulicatella | ASV140 | 1.157 (0.881) | 0.27 | 1.244 (0.983) | 0.28 |

^a^ Effect sizes and p-values of the comparison: controls versus lesional cases. ^b^ Effect sizes and p-values of the comparison: non-lesional cases versus lesional cases.
