# Supplementary material for: Composition of cutaneous bacterial microbiome in seborrheic dermatitis patients: A cross-sectional study
Source: PLoS One. 2021 May 24;16(5):e0251136. doi: 10.1371/journal.pone.0251136 (PMC8143393; doi:10.1371/journal.pone.0251136)
Supplement: S1 File — (DOCX) [file pone.0251136.s009.docx]

**S1 File.**

**MATERIALS AND METHODS**

**Bioinformatic pipeline**

Quality filtering was performed in DADA2 using the following criteria: trim=0, maxEE=c(2,2), truncQ=2, rm.phix=TRUE. Filtered reads were run through the DADA2 Amplicon Sequence Variant (ASV) assignment and ASVs were assigned a taxonomy from the SILVA version 132 rRNA database (1) using the RDP naïve Bayesian classifier (2). Data tables were combined into a phyloseq object using Phyloseq (3).

**Phylogenetic analysis**

For the phylogenetic analysis we used the MEGA-X software (4). This was done in several steps. First, we uploaded fasta sequence reads of the ASVs with suggestive differences in composition between lesional cases and controls into the software. Then, we aligned the sequences using the MUSCLE program (5) with neighbour joining method (6). Next, the phylogenetic tree was constructed using the aligned sequences using the Neighbour-joining method (6). The evolutionary distances were computed using the Maximum Composite Likelihood method (7). All ambiguous positions were removed for each sequence pair (pairwise deletion option). A phylogenetic tree can be interpreted as follows: branches (sequences) from the same node are more closely related to each other than branches from other nodes. The longer the branches the more differences between the sequences there are.

**RESULTS**

**Descriptive statistics of the microbiome data: Filtering steps**

After the sequencing and bioinformatic steps the table with amplicon sequence variants (ASVs) found in samples are imported into the package phyloseq for further downstream analysis. The total dataset consisted of a total of 15980 amplicon sequence variants identified in 895 samples, of which 25 were negative controls (air swabs taking during the sampling collection at the research centre).

We first excluded obvious contaminant phyla based on literature. These included *Planctomycetes*, *Epsilonbacteraeota*, *Cyanobacteria*, *Chloroflexi* , Ambiguous_taxa, *BRC1*, *WPS-2*, *FBP*, *Deinococcus*-*Thermus*, *Cyanobacteria* and *Epsilonbacteroraeota*. Further, we excluded samples with less than total 1000 reads, which were mostly negative controls. Next, we also removed a sample with an unusual high count since it is also likely to be the result on contamination. After this filtering steps we had 15111 ASVs in 533 samples including four negative controls, which were further removed. Finally, we used 5% prevalence filtering (8), meaning that we included ASVs that were present in at least 5% of all samples. This left a remaining of 185 taxa in 529 samples, of which 436 were controls, 71 were non-lesional cases and 22 were lesional cases. This dataset was then used for the downstream analysis described in the main manuscript.

**Analysis of alpha diversity**

Since measures of alpha diversity are sensitive to different library sizes, we estimated Chao1 and Shannon diversities in our dataset at different filtering thresholds, namely: minimal filtering (only contaminant phyla were left out); before 5% prevalence and after 5% filtering prevalence. In addition, we also rarefied the original dataset to have exactly 10000 counts per sample. For this we used the function 'rarefy_even_depth’ with sample size of 10000. S3-S5 figs showed the pairwise comparison of the Chao1 and Shannon diversity per lesional, non-lesional and control groups. In all cases the lesional cases showed the highest diversity confirming our results.

**REFERENCES**

1. Quast C, Pruesse E, Yilmaz P, Gerken J, Schweer T, Yarza P, et al. The SILVA ribosomal RNA gene database project: improved data processing and web-based tools. Nucleic Acids Research. 2012;41(D1):D590-D6.

2. Wang Q, Garrity GM, Tiedje JM, Cole JR. Naive Bayesian classifier for rapid assignment of rRNA sequences into the new bacterial taxonomy. Appl Environ Microbiol. 2007;73(16):5261-7.

3. McMurdie PJ, Holmes S. phyloseq: an R package for reproducible interactive analysis and graphics of microbiome census data. PLoS One. 2013;8(4):e61217.

4. Kumar S, Stecher G, Tamura K. MEGA7: Molecular Evolutionary Genetics Analysis Version 7.0 for Bigger Datasets. Mol Biol Evol. 2016;33(7):1870-4.

5. Edgar RC. MUSCLE: multiple sequence alignment with high accuracy and high throughput. Nucleic Acids Research. 2004;32(5):1792-7.

6. Saitou N, Nei M. The neighbor-joining method: a new method for reconstructing phylogenetic trees. Mol Biol Evol. 1987;4(4):406-25.

7. Tamura K, Nei M, Kumar S. Prospects for inferring very large phylogenies by using the neighbor-joining method. Proc Natl Acad Sci U S A. 2004;101(30):11030-5.

8. Dawson TL, Jr. Malassezia globosa and restricta: breakthrough understanding of the etiology and treatment of dandruff and seborrheic dermatitis through whole-genome analysis. J Investig Dermatol Symp Proc. 2007;12(2):15-9.
